# Interfering with Wnt signalling alters the periodicity of the segmentation clock

**DOI:** 10.1016/j.ydbio.2009.02.035

**Published:** 2009-06-01

**Authors:** Sarah Gibb, Anna Zagorska, Kristin Melton, Gennady Tenin, Irene Vacca, Paul Trainor, Miguel Maroto, J. Kim Dale

**Affiliations:** a Division of Cell and Developmental Biology, College of Life Sciences, University of Dundee, Dow Street, Dundee, DD1 5EH, Scotland, UK; b Stowers Institute for Medical Research, 1000 E 50th Street, Kansas City, MO, 64110, USA; c University of Kansas School of Medicine, 3901 Rainbow Boulevard, Kansas City, KS 66160, USA; d Cincinnati Children's Hospital Medical Center, Division of Neonatology 3333 Burnet Avenue, ML#7009 Cincinnati, OH 45229, USA

**Keywords:** Notch, Wnt, Embryo, Mouse, Chick, Somite, Segmentation clock

## Abstract

Somites are embryonic precursors of the ribs, vertebrae and certain dermis tissue. Somite formation is a periodic process regulated by a molecular clock which drives cyclic expression of a number of clock genes in the presomitic mesoderm. To date the mechanism regulating the period of clock gene oscillations is unknown. Here we show that chick homologues of the Wnt pathway genes that oscillate in mouse do not cycle across the chick presomitic mesoderm. Strikingly we find that modifying Wnt signalling changes the period of Notch driven oscillations in both mouse and chick but these oscillations continue. We propose that the Wnt pathway is a conserved mechanism that is involved in regulating the period of cyclic gene oscillations in the presomitic mesoderm.

## Introduction

The first overt sign of segmentation is seen very early in development in the paraxial mesoderm tissue. As development proceeds, this mesenchymal tissue becomes progressively segmented in an anterior to posterior direction as pairs of epithelial structures called somites bud off the anterior end of the unsegmented paraxial or presomitic mesoderm (PSM), with a periodicity that is species specific; in chick this takes 90 min, in mouse 120 min and in humans 8 h. The periodicity of this process is believed to be regulated by a segmentation clock that drives the periodic expression of a number of clock genes in the PSM of vertebrate embryos. The expression of these clock genes appears as a wave of transcription that sweeps across the PSM caudorostrally in a cyclical fashion with a periodicity that matches somite formation (Pourquie, 2003). The majority of clock genes identified so far belong to the Notch signalling pathway (reviewed in Dequeant and Pourquie, 2008; Kageyama et al., 2007; Rida et al., 2004). Studies aimed at elucidating the function of these clock genes have provided a wealth of data supporting an essential role for the Notch pathway in the mechanism of the segmentation clock (Bessho et al., 2003; Dale et al., 2003; Serth et al., 2003). Recent findings have implicated two other signalling pathways in the mechanism of the clock. A number of Wnt and FGF pathway members were shown to cycle in the mouse PSM (Aulehla et al., 2003; Ishikawa et al., 2004; Suriben et al., 2006; Wahl et al., 2007). Furthermore, recent reports have demonstrated that these three pathways interact reciprocally within the mechanism of the mouse segmentation clock (Aulehla et al., 2003; Dequeant et al., 2006; Dequeant and Pourquie, 2008; Ishikawa et al., 2004; Kawamura et al., 2005; Niwa et al., 2007; Wahl et al., 2007; Feller et al., 2008; Ozbudak and Pourquie, 2008).

To date there has been no report of cyclic PSM expression of Wnt related clock genes in any vertebrate species other than mouse. In order to address how conserved the regulation of the clock mechanism is, it is important to investigate whether components of the same pathways oscillate in different species. Another vitally important aspect of this segmentation clock which remains a mystery is the molecular mechanism regulating the period of these oscillations. The only studies addressing this issue to date suggested that the oscillation period does vary during development (Jouve et al., 2002), but it is completely unknown how this period is regulated.

## Materials and methods

### Embryos

White leg horn chicken (*Gallus gallus*) eggs were sourced from Henry Stewart & Co (Lincolnshire) and Winter Farm (Royston) and incubated at 38.5 °C in a humidified incubator. Embryos were staged according to the Hamburger Hamilton (HH) developmental table (Hamburger and Hamilton, 1992) and by somite counting. We used HH stage 12–15 embryos for the expression analysis of chick homologues of the Wnt pathway components that cycle in the mouse PSM. Wild-type CD1 mouse (*Mus musculus*) embryos were obtained from timed mated pregnant females between 9.5 and 10.5 days postcoitum (dpc).

### Cloning of Wnt target genes

Primers were designed to target sequences from the Ensembl database (www.ensembl.org) corresponding to either exonic or intronic sequences for each target gene. The primers were then used to perform PCRs to amplify target sequences which were then cloned into the pGEM-T Easy vector (Promega). All sequences were verified by restriction analysis and DNA sequencing. The following primers were used: chick exonic *Nkd1*, forward 5′-CTTTGCCTCCAGAGAAGACG-3′ and reverse 5′-TGGAGACTGGAAGGTTTTGC-3′; chick intronic *Nkd1*, forward 5′-AAGCAGAGCACATCCTCACA-3′ and reverse 5′-CTCCTTTGGGCTAGGTTTCC-3′; chick intronic *Axin2*, forward 5′-CCTTGAAGCTCCAAAGCAAC-3′ and reverse 5′-AAGCTGGGCCTCTCTGGTAT-3′; chick intronic *Lef1*, forward 5′-CCTTTTCTGCCTTGTTTTGC-3′ and reverse 5′-AAGGCCCAAGATTGAGTGTG-3′; mouse intronic *Lef1*, forward 5′-CGCTGGTAACCCGAGTAGAG-3′ and reverse 5′-TGTGGTTAATGGGAGGGAAA-3′.

### Chick explant culture

Embryos were dissected in PBS and then transferred to a microdissection dish containing L15 (Gibco) where the caudal half of the embryo was isolated. This explant was bisected down the midline such that each embryo provided two identical explants. Each explant was then positioned endoderm down on a 1.2 μM Isopore membrane filter (Millipore) and the filter floated on top of 500 μl of Wnt3a conditioned media or its appropriate control (kindly provided by D. Alessi, University of Dundee) or 500 μl culture media (Optimem (GIBCO) supplemented with 5% heat-inactivated FBS, 20 μM glutamine and 50 μg/ml gentamicin) with reagent added as indicated. Wnt3a conditioned media was collected as supernatant from cells transfected with control plasmid or plasmid encoding Wnt3a (McManus et al. 2005). Typically reagents used were 200 μM or 400 μM Casein Kinase Inhibitor (CKI-7) (USBiological), 25 μM SU5402 (Calbiochem), 0.1–100 μM γ-secretase inhibitor IX (DAPT) (Calbiochem), 10 mM LiCl, 4–10 μg/ml soluble frizzled receptor protein (SFRP2 R&D systems) or the corresponding control, 100% ethanol, dimethylsulphoxide (DMSO), 10 mM KCl or PBS, 1% BSA respectively. Alternatively, for the fix and culture experiments (Palmeirim et al. 1997), both explants were cultured for a minimum of 15 min and then one explant fixed immediately (4% formaldehyde in PBS, 2 mM EGTA) while the other was cultured for 1 h longer or the stated time. For fix and culture experiments performed to assess cycling activity in the presence of drug, both explants were cultured in either 200 μM or 400 μM CKI-7 or ethanol for 3 h and then one explant fixed immediately while the other was cultured for 45 min longer. Fix and culture of the older HH stage 22 tail explants was performed by culturing the explants in a hanging drop of culture medium rather than on a filter.

### Mouse culture

Mouse explants were prepared as previously described (Dale et al. 2006) whereby each embryo's caudal region was bisected down the mid-line and one side was cultured in a hanging drop of mouse media (DMEM (Gibco), 10% FBS, 1% penicillin–streptomycin, 10 ng/ml Fgf2 (PeproTech)) for the given time period in the presence of reagent at the stated concentration while the contralateral side of the same embryo was cultured in the appropriate control. Typically reagents used were 100 μM or 200 μM Casein Kinase Inhibitor (CKI-7) (USBiological), 100 μM γ-secretase inhibitor IX (DAPT) (Calbiochem), 25 μM SU5402 (Calbiochem) or the corresponding control, 100% ethanol or dimethylsulphoxide (DMSO) respectively. Alternatively, for the fix and culture experiments to assess cycling activity in the presence of CKI-7, both explants were cultured in either CKI-7 or ethanol.

### In situ hybridisation

Whole-mount in situ hybridisations utilising exonic probes were performed as described (Henrique et al., 1995). The following modifications to this protocol were used for intronic probe in situ hybridisations. Samples were hybridised with probe for 40 h in a low stringency hybridisation mix (50% formamide, 5 × SSC, 5 mM EDTA, 50 μg/ml tRNA, 0.2% Tween 20, 0.1% SDS, 100 μg/ml heparin) and post-hybridisation washes were performed in post-hybridisation buffer (50% formamide, 0.1% Tween 20, 1 × SSC). Samples were processed either by hand or using the InsituPro VS Robot (Intavis AG). The protocol was modified for the older HH stage tail explants to include 23 min proteinase K treatment and after the development of signal to the desired point, any background was washed out with whitening solution (40 g NaCl, 1 g KCl, 130.4 ml 1 M Tris/HCl pH7.8, 55 ml Tween 20, water to 500 ml). Embryos were then washed twice with PBT.

### Phospho-HistoneH3 staining

Fixed PSM explant tissue was proteinase K (Roche) treated and fixed (4% formaldehyde in PBS, 2 mM EGTA, 0.1% glutaraldehyde (Sigma)) prior to washing in PBST. Explants were then blocked in 2% BSA in PBST for 2 h at room temperature. Anti-phospho histone-H3 antibody (Upstate) was then added at 10 μg/ml and the samples incubated at 4 °C overnight. Specimens were then washed for 10 h in a minimum of three changes of PBST before the Alexa-fluor488 conjugated mouse anti-rabbit antibody (Invitrogen) was added at 2 μg/ml in PBST and left overnight at 4 °C. Samples were then washed for around 10 h in PBST with a minimum of 3 changes of solution. Explants were then mounted on SuperFrost microscope slides (VWR) using Hydromount (National Diagnostics) and stored at 4 °C prior to analysis for fluorescent signal using a compound fluorescence microscope (Leica DM5000 B). Fluorescent cells in the PSM were counted manually using the 40× objective. Results were analysed statistically using the paired *T*-Test. Results were also shown graphically by means of a boxplot to show the spread of the data, with the box displaying the middle half of the data. The line in the box shows the median.

### Apoptosis assay

The ApopTag kit (Chemicon) was used to perform a version of the TUNEL assay on chick PSM explants with the following modifications. Samples were prepared for the assay by proteinase K treatment and fixation (4% formaldehyde in PBS, 2 mM EGTA, 0.1% glutaraldehyde) prior to washing in PBST. Equilibration buffer was then added to the explants and left for 15 min at room temperature. Incubation in working strength terminal transferase (TdT) was carried out overnight at 4 °C and the reaction stopped with stop/wash buffer by incubation at 37 °C for 40 min. Specimens were then washed for a minimum of six times in TBST before heat inactivating the enzyme at 65 °C for 20 min. Samples were then incubated in blocking reagent (20% Blocking Reagent (Roche), 20% heat inactivated goat serum in TBST) for 2 h at room temperature before addition of 150 mU/ml anti-digoxigenin-AP antibody (Roche) and incubation overnight at 4 °C. Explants were then washed three times for 1 h in MABT (0.1 M maleic acid, 0.15 M NaCl, 10% Tween 20, pH 7.5) followed by two 10 minute washes in NTMT (100 mM NaCl, 100 mM Tris HCl pH 9.5, 50 mM MgCl_2_, 1% Tween 20). Samples were then incubated in NBT/BCIP colour reagent (0.027% 50 mg/ml NBT (Promega), 0.014% 50 mg/ml BCIP (Promega) in NTMT) for a minimum of 15 min until sufficient signal had developed. Specimens were analysed using a Leica MZ16 APO microscope using a Jenoptik camera. Labelled cells in the PSM were counted manually using the 40x objective. The positive cells in the PSM were blind counted, so that the person performing the manual count did not know which explant was treated with CKI-7 and which was the control in each pair. The results were analysed by paired *T*-Test. Results were also shown graphically by means of a boxplot to show the spread of the data, with the box displaying the middle half of the data. The line in the box shows the median.

### Western blot analysis

Caudal embryo extracts were prepared by pipetting treated tissue in lysis buffer (50 mM Tris pH 7.4, 0.27 M sucrose, 1 mM Na-orthovanadate pH 10, 1 mM EDTA, 1 mM EGTA, 10 mM Na-β-glycerophosphate, 50 mM NaF, 1% Triton-X 100, 0.1% β-mercaptoethanol) and centrifuging for 10 min at 4 °C, 16,000 RCF to pellet insoluble debris. Samples were then analysed by Bradford assay and 20 μg protein prepared for loading onto 4–12% Bis–Tris acrylamide gels (Invitrogen) using NuPAGE LDS sample buffer (Invitrogen) and NuPAGE sample reducing agent (Invitrogen). Gels were then blotted using standard molecular techniques and the resultant nitrocellulose membranes (Whatman) were blocked in 10% milk in TBS–0.25% Tween 20 (TBST). Membranes were treated with rabbit anti-phospho-β-catenin S33, S37, T41 antibody (cell signalling) or mouse anti-GAPDH antibody (Abcam) in 5% BSA in TBST, followed by secondary antibody (HRP) in 5% milk in TBST and standard ECL revelation (Pierce). Membranes treated with rabbit anti-phospho-β-catenin antibody were stripped using commercial stripping buffers as directed by the manufacturer (Chemicon) and then blocked again in 10% milk in TBST before briefly washing in TBST and adding mouse anti-total β-catenin antibody (Santa Cruz) at a 1:1000 dilution in 5% BSA in TBST. This was followed by secondary antibody and ECL as described above.

### BrdU assay

BrdU assays were performed on chick explants after treatment of the control side of each explant pair with DMSO or the treated side with 50 μM aphidicolin (Sigma) for 3.5 h in chick media (as above). Explants were then pulsed with 0.1 mM BrdU for 30 min by adding BrdU directly to the medium before fixing in fresh PFA overnight at 4 °C. Explants were then removed from the Millipore filters and washed in wash buffer (1% Triton, 1% Tween 20 in PBS) 3 times for 5 min and then 4 times for 30 min (wash buffer washes). Samples were then treated with 2 M HCl for 1 h at room temperature then washed for 20 min in 0.1 M Borax, pH 8.5. The above wash buffer washes were then repeated before dehydrating then rehydrating the explants in the following methanol wash sequence (methanol diluted in wash buffer) for 5 min per wash; 25% methanol, 50% methanol, 75% methanol, 100% methanol, 100% methanol, 75% methanol, 50% methanol, 25% methanol, 0% methanol. Explants were then washed 3 times for 5 min in wash buffer supplemented with 1% BSA (blocking solution). Explants were then blocked overnight at 4 °C in this solution with the addition of 10% goat serum (GIBCO) and then treated with mouse anti-BrdU antibody (Roche) at a 1:100 dilution in blocking solution containing goat serum for 4 nights. The series of wash buffer washes described earlier was then repeated followed by 3 times 5 minute blocking solution washes. The explants were then blocked overnight at 4 °C in blocking solution supplemented with 10% goat serum before adding Alexa-fluor488 conjugated rabbit anti-mouse antibody (Invitrogen) at a 1:250 dilution in blocking solution with goat serum overnight at 4 °C. The secondary antibody was then washed off by repeating the above wash buffer washes and the samples mounted using Hydromount (National Diagnostics). Explant images were captured using a compound fluorescence microscope (Leica DM5000 B) ensuring the same exposure time and magnification was used for all explant pairs.

## Results

### Chick homologues of the Wnt pathway components that cycle in mouse do not cycle in the chick PSM

**Fig. 1:**
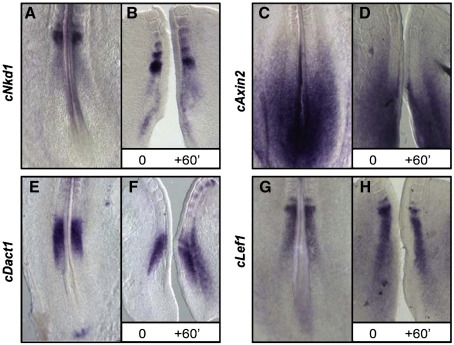
Wnt signalling components do not cycle in chick PSM. (A, G, H) Intronic and (B) Exonic probes to *cNkd1* and *cLef1* show a gradient of PSM expression. (B, H) Fix and culture assays, where explants of the caudal embryo are bisected down the midline, following which both sides are cultured for 15 min minimum and then one explant fixed while the other was cultured for 45 min longer, confirms absence of oscillatory expression for both genes. (C, D) Intronic *cAxin2* expression is restricted to the caudal PSM. (D) The fix and culture assays showed no dynamism in expression. (E, F) Exonic *cDact1* expression is restricted to the rostral PSM. (F) The fix and culture assays showed no dynamism in expression.

To address whether the Wnt signalling pathway plays a role in the clock mechanism in chick we cloned the chick homologues of three reported mouse Wnt clock genes, namely, *cNkd1, cAxin2* and *cDact1* all of which are downstream targets and negative regulators of Wnt signalling. The criteria we used to define oscillatory gene expression in the PSM were a caudal to rostral wave of RNA expression sweeping the extent of the PSM or alternatively a domain of synchronized and periodic on–off RNA expression in the caudal PSM. Using an exonic probe to *cNkd1* we observed a stable gradient of *cNkd1* transcript starting at the level of the forming somite and extending caudally (*n* = 13, data not shown). By our criteria we did not therefore observe any cyclic expression of *cNkd1*. In order to ensure we did not miss any cyclic RNA expression through masking of waves of expression by RNA stability, we also cloned the intronic *cNkd1* probe and observed a similar profile (*n* = 13, Fig. 1A). Moreover, an *in vitro* fix and culture assay did not reveal any oscillatory behaviour (*n* = 18, Fig. 1B). The only differences in *cNkd1* expression were restricted to the level of the forming somite (Fig. 1B). Thus, we find that surprisingly *Nkd1* expression differs markedly in chick and mouse (Ishikawa et al., 2004); our unpublished observations). *mAxin2* is the second Wnt target gene reported to oscillate in the mouse PSM and it has been described to oscillate out of phase with the Notch target clock genes (Aulehla et al., 2003). Our analysis of *cAxin2* expression in the PSM using exonic (*n* = 10) and intronic (*n* = 16) probes revealed an expression profile restricted to the caudal PSM and the adjacent lateral plate tissue but the rostral PSM band observed in mouse (Aulehla et al., 2003) is missing in chick (Fig. 1C). Once again, the fix and culture assay using an intronic *cAxin2* probe did not reveal any synchronized, periodic on–off oscillatory behaviour in the caudal expression domain (*n* = 6, Fig. 1D). Thus, in contrast to the mouse homologue, *cAxin2* does not appear to cycle in the chick PSM. *mDact1* is a third Wnt target gene reported to oscillate in the mouse PSM (Suriben et al., 2006). Our analysis of *cDact1* expression in the PSM using exonic probes revealed an expression profile restricted to the rostral PSM (Fig. 1E). Once again, the fix and culture assay did not reveal any synchronized, periodic on–off oscillatory behaviour in the rostral expression domain (*n* = 6, Fig. 1F). Thus, in contrast to the mouse homologue, *cDact1* does not appear to cycle in the chick PSM. To investigate further the contribution of the Wnt pathway to the mechanism of the segmentation clock we analysed the expression of *Lef1* in both the mouse and chick PSM. Lef1 is a key downstream effector of the Wnt pathway (Filali et al., 2002; Logan and Nusse, 2004). The use of both exonic and intronic probes showed that in the mouse, *mLef1* is expressed as a rostrocaudal gradient throughout most of the PSM with a distinct band of expression in the rostral most part of the tissue corresponding to the forming somite (*n* = 19, data not shown). We then examined the expression profile of *c-Lef1* in the chick PSM and found that the pattern of expression is very similar to that of *cNkd1*. The expression in the PSM is seen as a non-dynamic rostrocaudal gradient while expression in the very rostral region at the level of the forming somite varies from embryo to embryo (*n* = 31, Figs. 1G, H). Chick explants analysed for *cLef1* expression following the fix and culture assay confirmed this expression profile (*n* = 4, Fig. 1H). In summary, our expression analysis of four Wnt downstream targets and effectors in the chick PSM show that none of them oscillate in the manner of a clock gene.

### Notch regulates both Notch and Wnt target gene expression in the chick and mouse PSM

**Fig. 2:**
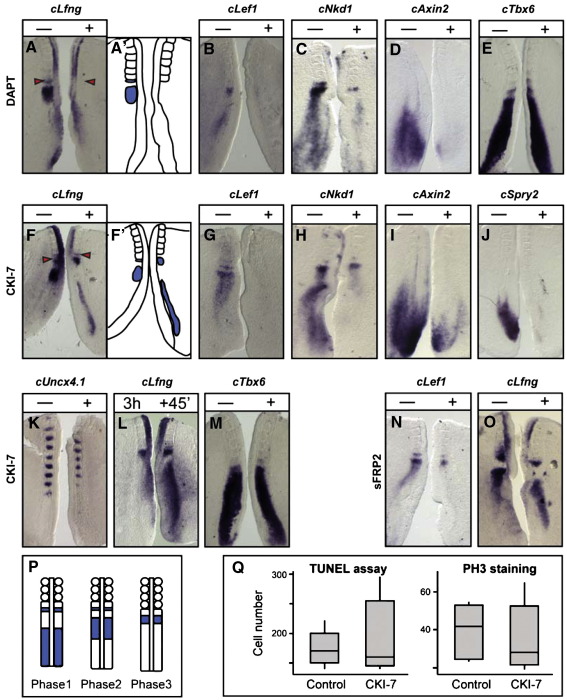
*cNkd1* and *cLef1* are Notch dependent and reduced Wnt signalling increases the periodicity of *cLfng* oscillations in the chick PSM. Chick explant pairs cultured for 3–4 h on a Millipore filter floating on 100 μM DAPT, 200 μM CKI-7 or 4–10 μM sFRP2 or control media supplemented with DMSO, PBS 0.1% BSA or ethanol respectively. Panels A′, F′ are schematic representations of panel A and panel F respectively showing *cLfng* expression in the PSM only. (A, A`) DAPT treatment led to loss of *cLfng* in the PSM. Neural tube expression was unaffected. (B) Expression of intronic *cLef1* was also lost. (C) Expression of *cNkd1* was also severely down regulated. (D) Expression of exonic *cAxin2* was also lost. (E) Exonic *cTBX6* expression was not affected. (F, F`) CKI-7 slowed the period of *cLfng*. Note the treated explant is phase 1 and the control is early phase 3 of the same cycle and has formed a new somite boundary. (G) CKI-7 led to loss of intronic *cLef1* expression. (H) Exonic *cNkd1* and (I) exonic *cAxin2* expression are severely down regulated on exposure to CKI-7. (J) Exonic *cSprouty2* expression is lost on exposure to CKI-7. (K) Exposure to CKI-7 led to the formation of fewer somites and stripes of *cUncx4.1* expression. (L) Explants showing cycling *cLfng* in 200 μM CKI-7 with the fixed explant showing phase 3 while the cultured explant has progressed to late phase 1. (M) Exonic *cTBX6* expression was not affected on exposure to CKI-7. (N) sFRP2 treatment down regulates *cLef1* expression. (O) The explant treated with sFRP2 is in early phase 2 and the control explant in early phase 3 of the *cLfng* cycle. (P) Schematic representation of clock gene expression during one oscillation in the PSM. (Q) Box plot showing spread of TUNEL staining measurements or phosphoH3 staining in explants treated with CKI-7 compared to untreated controls. Arrow heads show last somite boundary formed in culture. “+” indicates treated half and dashed line indicates control half.

**Fig. 3:**
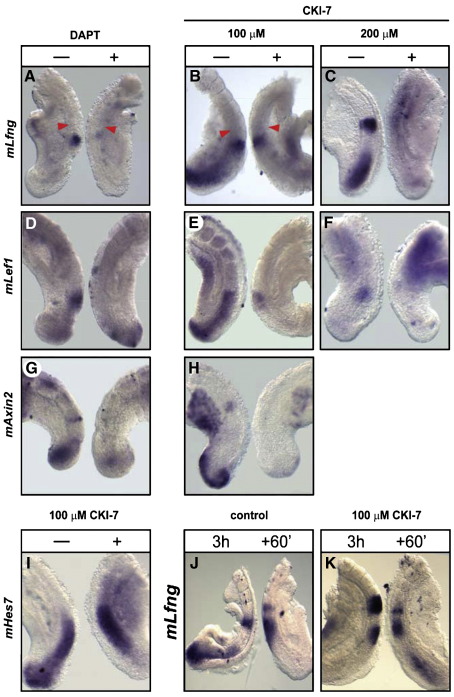
Notch and Wnt are co-dependent in mouse and Wnt regulates the periodicity of *mLfng* and *mHes7* oscillations in the mouse PSM. Caudal explant pairs of E9.5 mice were cultured for 4 h in the presence or absence of 100 μM DAPT, 100 μM or 200 μM CKI-7. (A) DAPT led to the severe down regulation of *mLfng* expression. (D, G) *mLef1* and *mAxin2* expression was also severely down regulated. (B) 100 μM CKI-7 slowed the period of *mLfng* in the mouse PSM. Note the control is in phase 1 and the treated explant is in phase 3 i.e. 1 phase behind the control. 100 μM CKI-7 down regulated (E) *mLef1* and (H) *mAxin2*. 200 μM CKI-7 severely down regulated (C) *mLfng* expression and (F) *mLef1* expression. Arrow heads show the last somite boundary formed in culture. (I) 100 μM CKI-7 slowed the period of *mHes7* oscillations. Note the control is in phase 1 and the treated explant is in phase 2 i.e. 2 phases behind the control. (J, K) Fix and culture assays of E9.5 explants where both sides are cultured either in CKI-7 or EtOH and analysed for *mLfng* expression. (J) Explants cultured in control media showing cycling *mLfng*. Fixed sample is in phase 1 while the cultured explant is in late phase 2. (K) Pair of explants both cultured in 100 μM CKI-7 showing dynamic *mLfng* expression. The fixed explant is in phase 3 while the cultured explant has progressed to early phase 1. “+” indicates treated half and dashed line indicates control half.

To investigate the hierarchy between Notch and Wnt signalling in the chick PSM we used a pharmacological approach and cultured chick caudal half embryo explants from two day old embryos *in vitro* for 4 h in the presence or absence of the Notch inhibitor DAPT, a dipeptidic γ-secretase inhibitor which selectively inhibits the cleavage of the Notch receptor at the membrane (Dale et al., 2003; Morohashi et al., 2006), and analysed expression of target genes from both pathways. Treatment with DAPT completely abolished expression of the Notch target gene *cLfng* in the PSM as expected (*n* = 21/21, Figs. 2A, A′). It is noteworthy that *cLfng* expression in the neural tube was unaffected. Surprisingly, we also observed a complete loss or severe down regulation of the Wnt target genes *cLef1* (*n* = 22/28, Fig. 2B) and *cNkd1* (*n* = 5/5, Fig. 2C) in the majority of DAPT treated explants while the other explant pairs were not affected. The Wnt target *cAxin2* was also severely down regulated in some explants following DAPT treatment (*n* = 9/28, Fig. 2D) while the majority of the other cases were not affected. In order to ensure the DAPT mediated defects were not due to a general disruption of PSM identity, patterning or integrity we looked at expression of the PSM marker *cTbx6*, which was unaffected along the length of the PSM (*n* = 13/13, Fig. 2E). Moreover, our analyses within a defined region of the PSM of either the number of phospho-histone H3 labelled cells or use of the TUNEL assay did not reveal any significant difference in either the number of proliferating cells (*n* = 5 explant pairs; *p* > 0.05) or the number of apoptotic cells (*n* = 5 explant pairs; *p* > 0.05) in treated versus control explants (data not shown). To allow a direct comparison with our chick data we then cultured E9.5 mouse caudal half embryo explants *in vitro* in the presence or absence of DAPT. This treatment led to a loss of *mLfng* expression as expected (*n* = 5/5, Fig. 3A), as well as a loss or severe down regulation of *mLef1* expression in the rostral PSM (*n* = 17/20, Fig. 3D). DAPT treatment also abolished *mAxin2* expression in the rostral PSM in 100% of treated explants and the caudal band was also lost in most explants but a proportion of the samples retained some restricted caudal expression in the PSM (*n* = 4/13, Fig. 3G). This residual expression in the caudal region of the PSM may be Notch independent and entirely Wnt dependent. Indeed, recent literature clearly indicates that the mechanism of gene regulation can differ greatly in the anterior and posterior PSM (Niwa et al., 2007). These data suggest that Notch signalling is required for the rostral progression of the cyclic expression of the Wnt-regulated gene *Axin2*. Expression of *mNkd1* following DAPT treatment appeared uniform and non dynamic along the PSM (*n* = 7/12, data not shown), which is consistent with published data (Ishikawa et al., 2004). Therefore, our data suggest that Notch signalling regulates expression of downstream components of the Wnt pathway in the PSM of both chick and mouse embryos.

### Wnt signalling regulates the periodicity of the segmentation clock in both mouse and chick

To allow a direct comparison of the effect of Wnt inhibition with the effects of Notch inhibition that we obtained from our *in vitro* data we used the same half embryo assay to investigate what effect Wnt attenuation has upon Notch target clock gene expression in the chick and mouse PSM. The first Wnt inhibitor used was CKI-7, an inhibitor of casein kinase 1 (Chijiwa et al., 1989; Price, 2006), which is a key positive regulator of the canonical Wnt pathway acting downstream of Dishevelled (Gao et al., 2002; Peters et al., 1999). Chick half embryo explants cultured for 4 h in the presence of CKI-7 (200 μM) led to a loss or severe down regulation of the PSM expression of a number of Wnt target genes, namely, *cLef1* (*n* = 33/34, Fig. 2G)*, cNkd1* (*n* = 6/9, Fig. 2H) and *cAxin2* (*n* = 6/7, Fig. 2I) as compared to the untreated control half. Thus we have severely down regulated expression of readouts of Wnt signalling along the entire anteroposterior axis of the PSM. CKI-7 treatment also led to a loss of expression of the FGF target gene *cSprouty2* in the PSM (*n* = 7/7, Fig. 2J). Following CKI-7 treatment exonic *cTbx6* expression in the PSM was unaffected in all cases, (*n* = 12, Fig. 2M). We did not observe any significant difference in either the number of proliferating cells (*n* = 5 explant pairs; *p* > 0.05) or the number of apoptotic cells (*n* = 5 explant pairs; *p* > 0.05) within a defined region of the PSM of CKI-7 treated versus control chick explants (Fig. 2Q).

Surprisingly, after 4 h of incubation with CKI-7 the domain of *cLfng* expression was 1–2 phases behind the phase of expression in the control non treated explant in all cases (*n* = 28, Figs. 2F, F′, P), and occasionally this resulted in the treated explant forming one less somite boundary than the control explant as judged by morphology or somite marker expression (*n* = 9/35, Figs. 2F, K). This data implies that CKI-7 treatment noticeably lengthens the period of the oscillations. To ensure that *cLfng* was still cycling we cultured both explants in CKI-7 for 3 h then fixed one half and cultured the other for another 45 min and we observed that *cLfng* does indeed continue to oscillate (200 μM CKI-7 [*n* = 4], Figs. 2L, P; 400 μM CKI-7 [*n* = 4], data not shown). To verify the extent to which the period had been extended we performed a time course of the fix and culture assay extending the time in culture for the cultured half until we determined the time needed for one full oscillation to occur such that the expression profiles matched in the two explants with an extra somite having formed in the cultured explant. Untreated control pairs completed one full oscillation and made an extra somite in 90 min (*n* = 7/8, Supplementary Fig. 1A), whereas explant pairs exposed to 200 μM CKI-7 did not complete a full oscillation in 90 min (*n* = 7/9, data not shown). Rather, we ascertained that exposure to 200 μM CKI-7 caused the oscillation period to become 115–120 min (*n* = 11/12, Supplementary Figs. 1B, C). Exposure to 200 μM CKI-7 also led to a slightly reduced level of *cLfng* expression as compared to untreated controls (*n* = 28, Figs. 2F, F′). Recent findings have shown that Notch signalling controls the synchronization of segmentation clock oscillations in zebrafish embryos and perturbation of Notch signalling will lead to asymmetric expression of cyclic genes (Ozbudak and Lewis, 2008). To ensure that observed asymmetry of *cLfng* expression was not a secondary effect of Wnt affecting Notch signalling levels we titrated the concentration of DAPT to which cultured explants were exposed to see if partial disruption of Notch signalling would lead to asymmetric *cLfng* expression (Table 1). Explants treated with 0.1 μM DAPT had no effect on *cLfng* expression as compared to untreated controls (*n* = 5/6, Supplementary Fig.2A — 1/6 showed faint rostral phase 3 band in the treated half). While the full repertoire of expression domains were seen in the control explants, explants treated with 1 μM DAPT showed no expression of *cLfng* along the PSM length although they all displayed a rostral phase 3 band of expression which was slightly weaker than in the control explant (*n* = 5/6, Supplementary Fig.2B — 1/6 had no PSM expression at all in the treated half). *cLfng* expression is more stable in this rostral phase 3 domain (Dale et al., 2003) and thus this domain of expression in treated samples may either reflect a delay/halt to the clock such that all explants are caught in phase 3 or alternatively this treatment may have blocked all new transcription and this rostral stripe may simply be due to residual expression. To test whether *cLfng* was still oscillating in these explants we cultured both explant halves for 3 h in 1 μM DAPT then fixed one explant and cultured the other for 45 min longer. In every case both sides were found to have a restricted domain of *cLfng* expression in the very rostral PSM indicative of the fact that *cLfng* is no longer cycling (*n* = 9, data not shown) compared to controls (*n* = 3, data not shown). Similarly some explants treated with 10 μM DAPT showed no expression of *cLfng* along the PSM length and only displayed a weak rostral band of expression while the full repertoire of expression domains was seen in the control explants (*n* = 3/7, Supplementary Fig.2C). The remaining samples treated with 10 μM DAPT did not show any PSM expression of *cLfng* at all (*n* = 4/7, Supplementary Fig. 2C). Explants treated with 100 μM DAPT also showed no PSM expression of *cLfng* at all (*n* = 25/25, Supplementary Fig. 2D). Thus, treatment with 0.1 μM, 1 μM, 10 μM or 100 μM DAPT did not lead to asymmetrical *cLfng* expression in any pair of explants analysed compared to the untreated control half. This titration curve covered all cases ranging from no effect on *cLfng* at 0.1 μM DAPT to complete loss of *cLfng* expression at 100 μM in the PSM. The intermediate doses did lead to lower levels of *cLfng* expression in the rostral PSM indicative of reduced Notch activity but this simultaneously led to a loss of cycling through the PSM. Thus, we conclude that the extension to the clock period that we observe following Wnt attenuation is unlikely to be due to an indirect effect of reduced Notch signalling activity levels.

To verify these observations we used an alternative means of attenuating Wnt signalling in the PSM, namely the soluble frizzled receptor protein (sFRP2), which acts via sequestering of endogenous ligand (for a review see Kawano and Kypta, 2003). As expected, explants cultured in the presence of sFRP2 led to a down regulation of *cLef1* expression in the treated explant as compared to the untreated control side (*n* = 5/9, Fig. 2N) (Olivera-Martinez and Storey, 2007), and this treatment also led to a prolonged period of *cLfng* oscillations (*n* = 5/11, Fig. 2O). As a means of further verification that the effects we observed following exposure to CKI-7 were due to inhibiting Wnt activity we used 10 mM LiCl or conditioned media from Wnt3a transfected cells in a rescue experiment. To that end we cultured explants in the presence or absence of CKI-7 alone or CKI-7 together with either Wnt3a conditioned media or LiCl, while the contralateral side was cultured in the appropriate control media. Again we found that 100% of explants exposed to 200 μM CKI-7 alone showed an extended period of the oscillations (*n* = 13/13). However, in a proportion of the explants cultured in the presence of both CKI-7 and either Wnt3a conditioned media (*n* = 8/16) or LiCl (*n* = 3/8) we saw a reversal of the CKI-7 effect such that the *cLfng* expression domain was either the same as the control side or even accelerated in the side treated with both CKI and Wnt3a conditioned media or LiCl (*n* = 3 the same; *n* = 5 accelerated for Wnt3a, *n* = 1 the same; *n* = 2 accelerated for LiCl, Supplementary Figs.3A, B and data not shown). Since this is never seen with CKI treatment alone, we interpret this effect as attributable to a rescue of the effects of CKI-7 by Wnt activation in the PSM, which supports the idea that in this context CKI-7 acts as an inhibitor of Wnt signalling and that Wnt may play a role in regulating the periodicity of clock gene oscillations in the chick PSM.

**Fig. 4:**
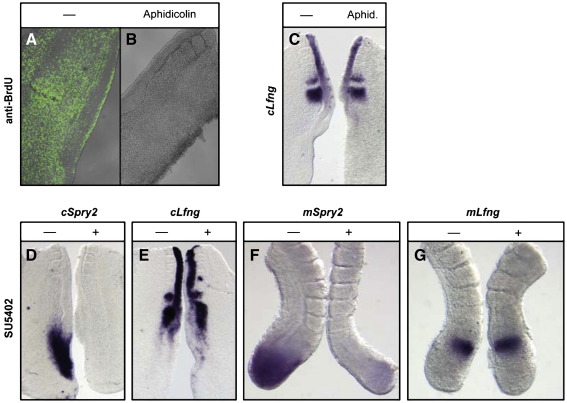
Aphidicolin treatment and inhibition of FGF signalling do not affect the periodicity of *Lfng* oscillations. Chick explants cultured in the presence or absence of 50 μM aphidicolin for 3.5 h and analysed for BrdU incorporation or *cLfng* expression. (A) Control half explant showing BrdU incorporation throughout the explant. (B) Aphidicolin treated explant showing no BrdU incorporation. (C) Pair of explants showing the same phase of *cLfng* expression in the presence and absence of aphidicolin. (D, E) Chick explant pairs cultured in 25 μM SU5402 or control media supplemented with DMSO. (D) SU5402 treatment led to loss of *cSprouty2* in the PSM. (E) Expression of *cLfng* was unaffected. (F, G) Mouse explant pairs cultured for 3 h 50 min in 25 μM SU5402 or control media supplemented with DMSO. (F) SU5402 treatment led to loss of *mSprouty2* in the PSM. (G) Expression of *mLfng* was unaffected. “+” indicates treated half and dashed line indicates control half.

To rule out the possibility that the observed effect of attenuated Wnt signalling was due to an effect on cell cycle we cultured chick half embryo explants in the presence or absence of aphidicolin, thereby blocking entry into S-phase. A four hour culture in the presence of this drug prevented BrdU incorporation in the treated explant as compared to controls (*n* = 6/6, Figs. 4A, B). Under these conditions *cLfng* expression was unaffected (*n* = 11/13, Fig. 4C). Thus, we conclude that the extension to the clock period that we observe following exposure to CKI-7 is not due to an indirect effect of reduced Wnt on cell proliferation.

In order to investigate if Wnt signalling plays a similar role during mouse development we treated E9.5 mouse half embryo explants with CKI-7. Following exposure to 100 μM CKI-7, *mLef1* (*n* = 9/15, Fig. 3E) and *mAxin2* (*n* = 5/6, Fig. 3H) expression was down regulated in the PSM. As in chick, we observed that the oscillation period of both *mLfng* (*n* = 11/22, Fig. 3B) and *mHes7* (*n* = 7/12, Fig. 3I) was extended. Under these conditions *mLfng* expression continued to be dynamic as judged by culturing both explants in the presence of 100 μM CKI-7 for 3 h following which one explant was fixed immediately while the other was cultured for 60 min longer (*n* = 6/6, Figs. 3J, K). However, exposure to 200 μM CKI-7, as used in chick, severely down regulated PSM expression of *mLfng*, *mHes7* and *mLef1* (*n* = 23/28, *n* = 5/5 and *n* = 11/14, respectively, Figs. 3C, F). Under these conditions *mLfng* was no longer dynamic in the PSM as judged by the fix and culture assay with both explants cultured in the presence of 200 μM CKI-7 (*n* = 4/4, data not shown). In summary, these data suggest a novel conserved role for Wnt signalling in the regulation of the period of clock gene oscillations in chick and mouse, and secondly, they indicate that Wnt signalling regulates expression of the Notch target clock gene, *Lfng*, in both chick and mouse.

### Inhibition of FGF signalling does not affect the periodicity of the segmentation clock

The expression of *FGF8* in the caudal PSM of mice and chick embryos is known to be regulated by Wnt signalling (Aulehla et al., 2003). A caudorostral gradient of expression of both *FGF8* and *Wnt3a* has been proposed to both maintain cells of the caudal PSM in an undetermined immature state and, by interacting with clock gene oscillations, to also determine the size of somites. To investigate whether FGF8 signalling may mediate the effect of Wnt on the period of *cLfng* oscillations we cultured chick and mouse half embryo explants *in vitro* in the presence or absence of the FGF receptor 1 inhibitor SU5402 (Dubrulle et al., 2001; Olivera-Martinez and Storey, 2007), and analysed expression of *Lfng* and the FGF target gene *Sprouty2*. Treatment with 25 μM SU5402 completely abolished expression of *cSprouty2* in the chick PSM as expected (*n* = 4/4, Fig. 4D, Table 1). However, exposure to SU5402 had no effect on *cLfng* expression (*n* = 9/9, Fig. 4E). Similarly, treatment with SU5402 severely down regulated expression of *mSprouty2* in the mouse PSM as expected (*n* = 5/5, Fig. 4F) and had no effect on *mLfng* expression (*n* = 17/17, Fig. 4G). These data suggest that the role for Wnt signalling in regulating the clock period in the chick and mouse PSM is not mediated by FGF signalling.

### Wnt3a expression becomes down regulated in the tail bud prior to an increase in the periodicity of both clock gene oscillations and somite formation

**Fig. 5:**
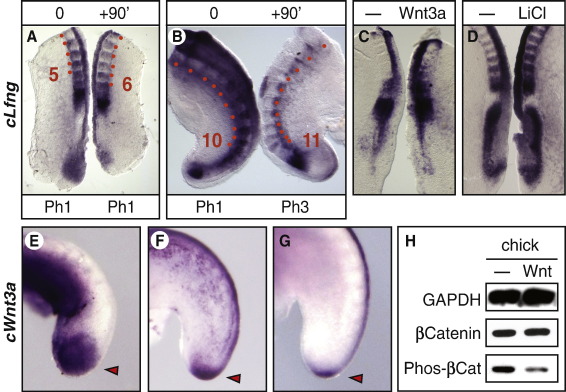
The periodicity of *cLfng* oscillations slows later in development following dramatic downregulation of *cWnt3a* expression in the tail bud. (A, B) Fix and culture assays performed on chick explants at different stages of development. (A) Two day old explants showing progression through one complete cycle of *cLfng* and formation of an extra somite within 90 min in the cultured half. Both explants show onset of phase I. (B) Chick explants dissected from HH stage 22 showing progression from phase I on the fixed side to phase III on the cultured side within 90 min i.e. the cultured explant does not complete a full oscillation in this time period. (C, D) Chick explant pairs cultured in Wnt3a conditioned media, 10 mM LiCl, control media alone or control media supplemented with KCl respectively. (C) Treatment with Wnt3a media did not affect the oscillation period of *cLfng*. (D) Treatment with LiCl did not affect the oscillation period of *cLfng*. (E–G) HH stage 20–23 chick embryos hybridised for *cWnt3a* expression showing broad expression in the tail bud at HH stage 20 (E) becomes dramatically reduced (F, G). (H) Western blot analysis where each lane contains protein extracted from the PSM of 5 E2 chick embryos cultured in control or Wnt3a media. Top row shows GAPDH as loading control. Middle row shows total β-catenin. Bottom row shows β-catenin when phosphorylated on the three residues targeted by GSK3β (S33, S37-P, T41-P). Dashed line indicates control. Red dots demarcate somites. Arrow heads highlight tail bud expression domain of Wnt3a.

We next looked to see if the periodicity of clock gene oscillations and somitogenesis changes during development and if this might be associated with a change in Wnt signalling. By analysing somite formation time in embryos in ovo during development, we observe that while the periodicity of somitogenesis in chick remains constant at 90 min throughout most of axis elongation, during development of the last somites in the tail bud of HH stage 22–24 chick embryos we observe an increase in the periodicity of somite formation (GT, JKD, MM; unpublished results). Comparable studies in a variety of other vertebrate embryos have reported a similar increase in the periodicity of somitogenesis in the late tail bud (Gomez et al., 2008; Schroter et al., 2008). Our data show that, in chick, this change in period of somite formation is matched by an extension in the period of clock gene oscillations, as judged by *cLfng* expression in fix and culture explants of these stages compared to explants dissected from younger embryos (*n* = 7/9 HH stage 22–23, and *n* = 9/10 HH stage 12 respectively, Figs. 5A, B). Thus, whereas a half embryo sample from a younger embryo cultured for 90 min exhibits an identical expression pattern to that in the non cultured half, this is not true of the samples from older embryos since they did not complete one full oscillation after this period of time in culture. Importantly, we also observed that *cWnt3a* is expressed in a broad area of the tail bud until HH stage 20, just prior to the observed slow down of somitogenesis and clock gene oscillations, whereupon *cWnt3a* expression is dramatically reduced to the very tip of the tail bud until it finally disappears at around HH stage 24 (Figs. 5E–G; Cambray and Wilson, 2007). These findings suggest that reduced Wnt signalling in the tail bud at these late stages contributes to a slow down in both the period of *cLfng* oscillations and somite formation.

### Exogenous Wnt activation does not shorten the period of the segmentation clock

We then performed the converse experiment and tested the effect of exogenous Wnt activity on the periodicity of the segmentation clock. To do this we cultured half embryo explants for 4 h in conditioned media from Wnt3a transfected cells (to ectopically activate the pathway in chick or mouse PSM). As expected, exposure to Wnt3a conditioned media reduced the amount of GSK3β mediated phosphorylation of β-catenin in the chick and mouse PSM (*n* = 5 and *n* = 5 respectively, Fig. 5H and data not shown) indicating the Wnt3a media is activating the canonical Wnt pathway in this tissue. However, under these conditions, the *cLfng* oscillation cycle was unaffected in the majority of samples (chick [*n* = 13/17], Fig. 5C; mouse [*n* = 7/7], data not shown) and was accelerated in only a few cases in the treated side compared to the control (chick [*n* = 4/17], data not shown). We repeated this assay using 10 mM LiCl to activate the Wnt signalling pathway and again observed that in the majority of cases the *Lfng* oscillation cycle was unaffected (chick [*n* = 12/15]; mouse [9/11], Fig. 5D and data not shown) despite clear activation of the Wnt signal transduction pathway (Fig. 5H and data not shown).

## Discussion

Our data provides evidence of clear conservation between mouse and chick, of some important features of the segmentation clock. Thus, we propose a critical, conserved and novel role for Wnt in the oscillator mechanism such that, in both mouse and chick, Wnt signalling regulates the periodicity of the segmentation clock. Moreover, we find that Notch and Wnt signalling co-regulate levels of expression of some of their respective target genes in the PSM of both mouse and chick. The results also provide the first demonstration of a clear species difference in the regulation of the segmentation clock in the PSM of the chick and the mouse such that while Notch target genes cycle in both species, we find that the Wnt regulated target genes *Nkd1*, *Dact1* and *Axin2* do not cycle across the chick PSM although they do oscillate in the mouse PSM (Aulehla et al., 2003; Ishikawa et al., 2004; Suriben et al., 2006, our unpublished data).

The published data in the field reports that expression of *mNkd1*, *mDact1* and *mAxin2* in the mouse PSM is Wnt dependent (Aulehla et al., 2003; Ishikawa et al., 2004; Suriben et al., 2006), although it is noteworthy that Notch is required for the expression of *mNkd1* to be dynamic in the mouse (Ishikawa et al., 2004). Our data looking at the expression of the chicken homologues of these three genes together with *cLef1*, using both exonic and intronic probes, show they do not cycle along the PSM of the chick embryo. We do not observe any expression of *cNkd1* or *cDact1* in the caudal PSM. Rather we see them expressed as a gradient in the rostral PSM and the same is true for *cLef1*. In contrast, *cAxin2* is solely expressed in the caudal PSM. These data suggest that these genes are unlikely to be key components of the avian molecular clock. It is noteworthy that the combined expression of these four genes (*cNkd1*, *cDact1*, *cLef1* and *cAxin2*) covers the whole of the PSM suggesting that although these Wnt components do not oscillate they reveal that Wnt signalling could in principle be present along the length of this tissue. Moreover, the position in the PSM where the rostral and caudal expression domains meet appears to match that of the proposed position of the determination front, which may indicate a switch in the output and role of Wnt signalling in distinct regions of the PSM. Since we have only looked at three outputs of Wnt signalling, the possibility remains that different components of this pathway could oscillate in the chick PSM or that in fact some subtle fluctuations in intensity do occur within the expression domains of these three genes and which would require more sensitive means of analyses to be identified. However, the fact that Notch driven clock gene oscillations continue when we inhibit Wnt signalling in the chick PSM, suggests that the Wnt pathway is not a critical oscillatory component of the avian clock mechanism.

The regulation of clock gene oscillations by the Notch signalling pathway has been demonstrated to play a key role in the molecular mechanism of the segmentation clock in a number of different vertebrate species (Rida et al., 2004). However, a role for the Wnt signalling pathway in the clock mechanism has so far only been highlighted in the mouse PSM (Aulehla et al., 2003; Ishikawa et al., 2004; Nakaya et al., 2005). It has been reported that Wnt signalling lies upstream of Notch in the mouse segmentation clock (Aulehla et al., 2003; Nakaya et al., 2005), although some studies have reported that these pathways can also act reciprocally (Ishikawa et al., 2004). Our data are in part consistent with Wnt signalling regulating Notch signalling, since inhibition of Wnt signalling reduces levels of expression of *Lfng* in both the mouse and chick PSM. However, in the mouse PSM inhibition of Wnt signalling causes a more severe down regulation of *mLfng* expression than in the chick and even leads to loss of *mLfng* oscillations. Moreover, the results suggest a more complex set of regulatory interactions since, in both chick and mouse, inhibition of Notch disrupts expression of *Lef1*, *Axin2* and *Nkd1,* as well as *Lfng* in the PSM and thus, the Notch pathway is not downstream but in fact regulates components of the Wnt pathway in both species. One possible explanation for this discrepancy between our data and those reports suggesting Wnt is upstream of Notch in the clock mechanism and therefore not affected by absence of Notch signalling (Aulehla et al., 2003; Nakaya et al., 2005) is that we adopted a pharmacological approach using a pan-Notch signalling inhibitor whereas the published reports look at the expression of Wnt target gene expression in mouse Notch mutants that are likely to retain some degree of Notch signalling since they also retain low levels of Notch target gene expression, such as *mLfng* (Morales et al., 2002). Thus, in summary our current data support a model in which the Notch and Wnt signalling pathways interact reciprocally in both the chick and mouse PSM (Ishikawa et al., 2004).

In recent years there has been a wealth of data provided in different vertebrate species as to the various molecular components involved in the segmentation clock mechanism (Rida et al., 2004; Ozbudak and Pourquie, 2008). However, one outstanding area we have little if any insight into is the means by which the periodicity of the molecular clock is set. This is of paramount importance since the interplay between the periodicity of the clock and the period of the regressing wavefront determines the number of cells allocated to each somite and thus ultimately regulates somite size. Moreover, for reasons yet to be determined the periodicity of the clock is an intrinsic and unique defining property of the different vertebrate species. Our data looking at the effect of Wnt inhibitors on *Lfng* oscillations in the PSM indicate a conserved role for the Wnt pathway in regulating the periodicity of the segmentation clock oscillations in both chick and mouse. In chick at least, it is well established that the pace of oscillations slows down in the rostral PSM (Palmeirim et al. 1997). Aulehla et al. (2008) reveal that this is a domain where nuclear B-catenin is absent and they suggest that the eventual slow down and arrest of clock oscillations in the rostral limit of the PSM under normal conditions require down regulation of Wnt signalling. In this study our data is entirely consistent with these predictions and we show that indeed a down regulation of Wnt signaling slows the pace of these oscillations. It will be very interesting to address whether this conserved novel role for Wnt extends to other vertebrate species.

It is striking to note that we were unable to shorten the period of the segmentation clock in either mouse or chick despite clear activation of the signal transduction pathway using Wnt3a conditioned media or LiCl. Two recent studies used conditional deletion of the destabilization domain of the endogenous mouse β-catenin to elevate Wnt signalling in the paraxial mesoderm (Aulehla et al., 2008; Dunty et al., 2008). Consistent with our observations, they report that constitutive activation of Wnt signalling did not affect the periodicity of *mLfng* oscillations using a live reporter for *mLfng* expression. These data are very similar to ours suggesting that the system cannot go any faster even in the presence of extra Wnt. An integral component in the regulation of the periodicity of clock gene oscillations must rely on the biochemistry of turnover of the clock gene mRNAs and proteins. Thus, our data following Wnt activation may be a reflection of the physical constraints on the maximum speed at which these regulatory components of the clock mechanism can be synthesized and metabolised within the embryonic tissue.

The potential role for Wnt signalling that we have reported here appears to be separable from previous roles attributed to this signalling pathway in the PSM. Both Wnt3a and FGF8 regulate the wavefront of maturation that acts in the caudal PSM to maintain cells in an immature, non determined state. Exogenous FGF or prolonged β-catenin stability, leads to a developmental delay in these cells (Dubrulle et al., 2001; Aulehla et al., 2008). Since, by attenuating Wnt signalling, we are performing the converse experiment, the effect on *Lfng* oscillations we observe is very unlikely to be linked to a more general maturation delay. Moreover, our data strongly suggest that the extended period of the clock cycle that we observe following Wnt attenuation is unlikely to be due to an indirect effect on cell proliferation. The role of Wnt3a in both PSM maturation and somite boundary positioning are believed to be mediated by FGF8 (Aulehla et al., 2008; Dunty et al., 2008; Dequeant and Pourquie, 2008; Kageyama et al., 2007; Rida et al., 2004). Consistent with these data we see that CKI-7 treatment causes a loss of the FGF target *cSprouty2* suggesting that FGF activity is also regulated by Wnt in the chick PSM. However, we demonstrate that the periodicity of *Lfng* oscillations is not affected in either the mouse or chick PSM in the presence of the FGF inhibitor SU5402 (Fig 4D–G; Dubrulle et al., 2001). This suggests that the role for Wnt signalling in regulating the clock period in the mouse and chick PSM is not mediated by FGF and is separable from its role in the regulation of the maturation wavefront. These data conflict with a study in mouse (Wahl et al., 2007). One possible explanation for this discrepancy is that those experiments were performed using 100 μM SU5402, whereas we used 25 μM SU5402 and since we also saw a loss of *cLfng* oscillations following exposure to 100 μM SU5402 (S.G. M.M, K.D unpublished observations), the effects on *cLfng* expression previously reported in the mouse may have been due to using a higher dose of inhibitor.

Previous reports in the literature have highlighted different levels of crosstalk between the Notch and Wnt pathways (reviewed in Hayward et al., 2008). Our data identifies an intriguing form of crosstalk between these two pathways. An important aspect will be to identify at what level of the signal transduction pathways this crosstalk between Notch and Wnt occurs in the PSM of mouse and chick and how this becomes translated into a change in the period of clock gene oscillations. One possibility is that this interplay is involved in regulating the stability of one or more of the key clock components. A better understanding of this interaction will be of great relevance and impact for both the understanding of somitogenesis and for the formulation of predictive models which attempt to simulate the process.

## Figures and Tables

**Table 1:** Calibration data to determine the lowest effective dose for the Notch signalling inhibitor DAPT, the Wnt signalling inhibitor CKI-7, the FGF signalling inhibitor SU5402 as judged by effective and reproducible loss of target gene expression in the PSM.

DAPT	0.1 μM	1 μM	10 μM	100 μM
*cLfng*	No effect (5)	Weak phase 3 domain (5)	Weak phase 3 domain (3)	No expression in PSM (25)
	Weak phase 3 domain (1)	No expression in PSM (1)	No expression in PSM (4)	

CKI-7	100 μM	150 μM	200 μM	400 μM

*cLef1*		No effect (11)	Severe down regulation (23)	Severe down regulation (10)
		Strong down regulation (5)	No effect (1)	
		Weak down regulation (6)		
*mLef1*	Severe down regulation (9)	weak down regulation (3)	Severe down regulation (11)	
	No effect (6)		No effect (3)	

SU5402	12.5 μM	25 μM	50 μM	100 μM

*cSprouty2*	Some weak expression (4)	No expression (8)	No expression (4)	No expression (4)
	No expression (1)			

## Appendix A Supplementary data

Supplementary Figure 1 CKI-7 treatment increases the periodicity of *cLfng* oscillations from 90 min to approximately 115–120 min in two day embryos. (A, B, C) Fix and culture assays performed on chick explants where both sides are initially cultured for 2.5 h in media supplemented with either ethanol or 200 μM CKI-7 following which one side is fixed while the other is cultured further for a specific period of time. (A) Following 2.5 h culture in media supplemented with ethanol one explant is cultured further for 90 min and it completes a full oscillation and forms an extra somite in this time period. Note both explants show phase 3 which shows progression through one complete cycle of *cLfng* and formation of an extra somite. (B) Following 2.5 h culture in 200 μM CKI-7 one explant is cultured further for 90 min and it does not complete a full oscillation in this time period. Note the fixed sample is an early phase 3 and the cultured sample is a late phase 1 of the next cycle. (C) Following 2.5 h culture in 200 μM CKI-7 one explant is cultured further for 120 min. Both explants show early phase 3 which shows progression through one complete cycle of *cLfng* and formation of an extra somite.

Supplementary Figure 2 Reducing levels of Notch signalling does not change the periodicity of *cLfng* oscillations. Chick explant pairs cultured in 0.1 μM DAPT, 1 μM DAPT, 10 μM DAPT or 100 μM DAPT, or control media supplemented with DMSO. (A) 0.1 μM DAPT treatment did not affect *cLfng* expression in the PSM. (B) 1 μM DAPT treatment led to a reduction in *cLfng* expression such that only a band of residual expression remained in the phase 3 domain. (C, D) 10 μM DAPT treatment led to either (C) a loss of *cLfng* expression throughout most of the PSM leaving only a band of residual expression in the phase 3 domain or (D) a total loss of *cLfng* expression throughout the PSM. (E) 100 μM DAPT treatment led to a total loss of *cLfng* expression throughout the PSM. Neural tube expression was unaffected.

Supplementary Figure 3 Wnt3a conditioned media and LiCl can reverse the effects of CKI-7. Chick explant pairs cultured for 3 h in the presence of both 200 μM CKI-7 and either Wnt3a conditioned media or 10 mM LiCl, compared to the control side which was cultured in either control conditioned media supplemented with EtOH or control media supplemented with KCl and EtOH and analysed for *cLfng* expression. (A) Wnt3a conditioned media rescued the effect of CKI-7 and accelerated the period of *cLfng* compared to the control. Note the control is in phase 1 and the treated explant is in phase 2 i.e. 1 phase ahead of the control. (B) LiCl rescued the effect of CKI-7 and accelerated the period of *cLfng* compared to the control. Note the control is in phase 1 and the treated explant is in phase 2 i.e. 1 phase ahead of the control.
