# Supplementary figures and images for: Interfering with Wnt signalling alters the periodicity of the segmentation clock
Source: Dev Biol. 2009 Jun 1;330(1):21–31. doi: 10.1016/j.ydbio.2009.02.035 (PMC2686089; doi:10.1016/j.ydbio.2009.02.035)

# Gibb\_Supp Fig1

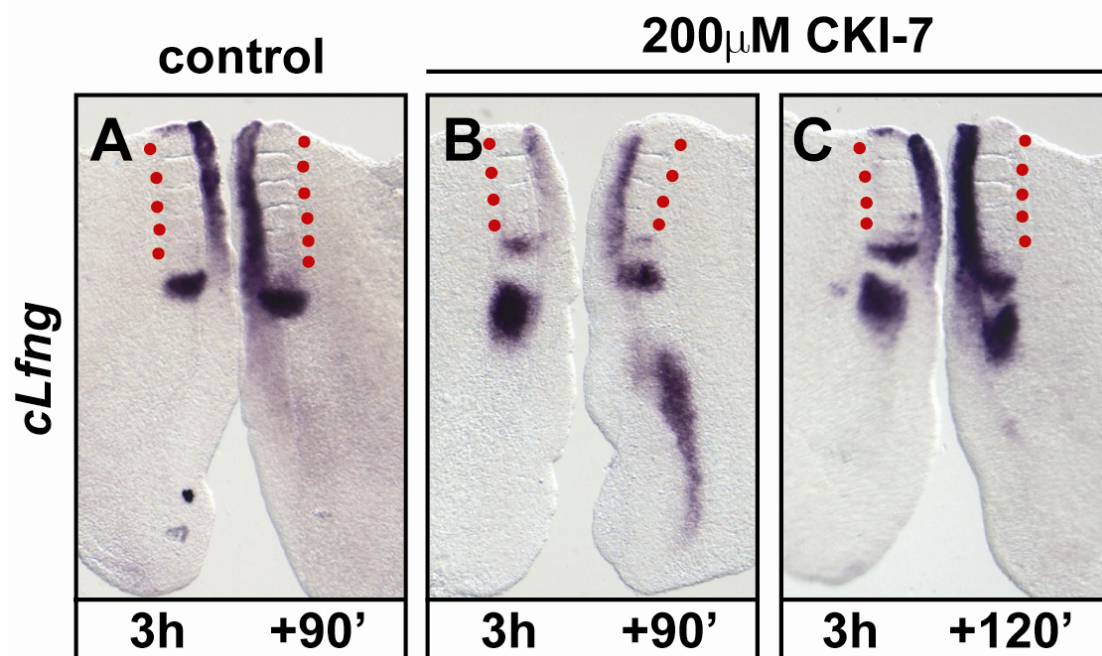

Supplement: Supplementary Figure 1 — CKI-7 treatment increases the periodicity of cLfng oscillations from 90 min to approximately 115–120 min in two day embryos. (A, B, C) Fix and culture assays performed on chick explants where both sides are initially cultured for 2.5 h in media supplemented with either ethanol or 200 μM CKI-7 following which one side is fixed while the other is cultured further for a specific period of time. (A) Following 2.5 h culture in media supplemented with ethanol one explant is cultured further for 90 min and it completes a full oscillation and forms an extra somite in this time period. Note both explants show phase 3 which shows progression through one complete cycle of cLfng and formation of an extra somite. (B) Following 2.5 h culture in 200 μM CKI-7 one explant is cultured further for 90 min and it does not complete a full oscillation in this time period. Note the fixed sample is an early phase 3 and the cultured sample is a late phase 1 of the next cycle. (C) Following 2.5 h culture in 200 μM CKI-7 one explant is cultured further for 120 min. Both explants show early phase 3 which shows progression through one complete cycle of cLfng and formation of an extra somite. [file mmc1.pdf]

Gibb\_Supp Fig2

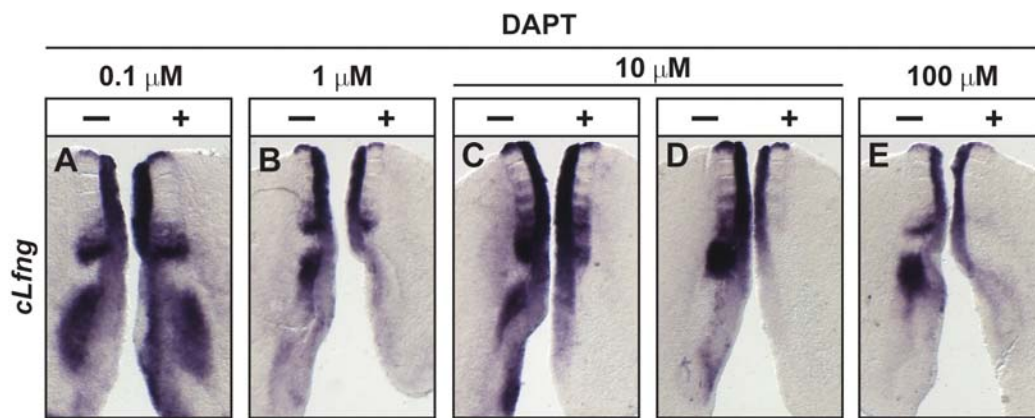

Supplement: Supplementary Figure 2 — Reducing levels of Notch signalling does not change the periodicity of cLfng oscillations. Chick explant pairs cultured in 0.1 μM DAPT, 1 μM DAPT, 10 μM DAPT or 100 μM DAPT, or control media supplemented with DMSO. (A) 0.1 μM DAPT treatment did not affect cLfng expression in the PSM. (B) 1 μM DAPT treatment led to a reduction in cLfng expression such that only a band of residual expression remained in the phase 3 domain. (C, D) 10 μM DAPT treatment led to either (C) a loss of cLfng expression throughout most of the PSM leaving only a band of residual expression in the phase 3 domain or (D) a total loss of cLfng expression throughout the PSM. (E) 100 μM DAPT treatment led to a total loss of cLfng expression throughout the PSM. Neural tube expression was unaffected. [file mmc2.pdf]

## Gibb\_Supp Fig3

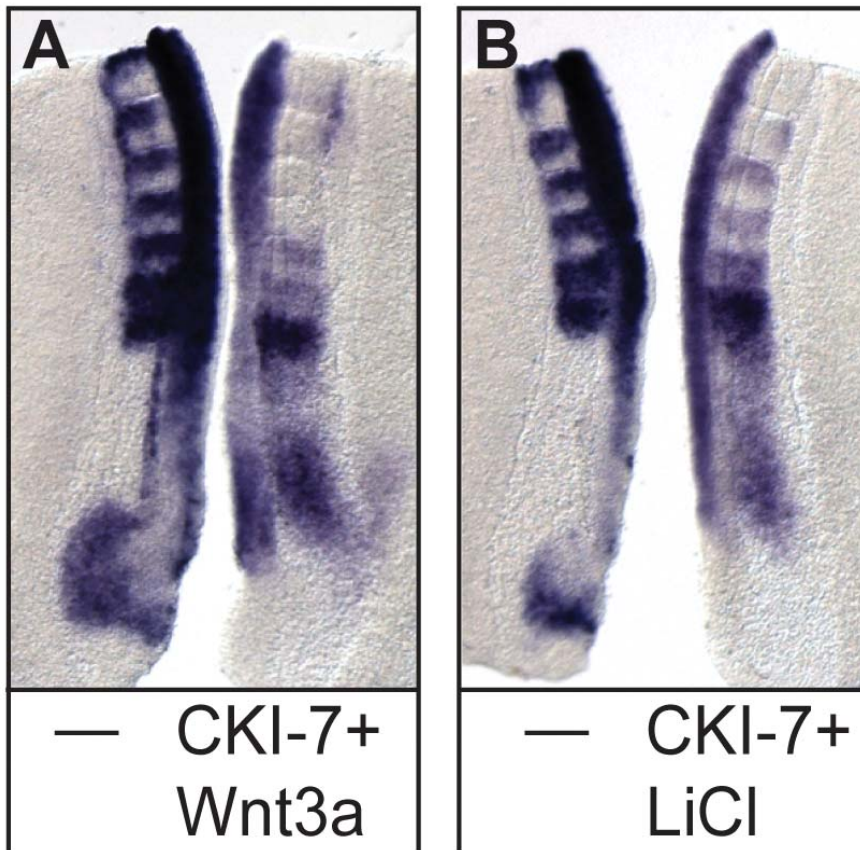

Supplement: Supplementary Figure 3 — Wnt3a conditioned media and LiCl can reverse the effects of CKI-7. Chick explant pairs cultured for 3 h in the presence of both 200 μM CKI-7 and either Wnt3a conditioned media or 10 mM LiCl, compared to the control side which was cultured in either control conditioned media supplemented with EtOH or control media supplemented with KCl and EtOH and analysed for cLfng expression. (A) Wnt3a conditioned media rescued the effect of CKI-7 and accelerated the period of cLfng compared to the control. Note the control is in phase 1 and the treated explant is in phase 2 i.e. 1 phase ahead of the control. (B) LiCl rescued the effect of CKI-7 and accelerated the period of cLfng compared to the control. Note the control is in phase 1 and the treated explant is in phase 2 i.e. 1 phase ahead of the control. [file mmc3.pdf]
